# Evaluation of machine learning models for predicting TiO_2_ photocatalytic degradation of air contaminants

**DOI:** 10.1038/s41598-024-64486-7

**Published:** 2024-06-13

**Authors:** Muhammad Faisal Javed, Muhammad Zubair Shahab, Usama Asif, Taoufik Najeh, Fahid Aslam, Mujahid Ali, Inamullah Khan

**Affiliations:** 1 Department of Civil Engineering, Ghulam Ishaq Khan Institute of Engineering Sciences and Technology, Topi, Pakistan; 2 Western Caspian University, Baku, Azerbaijan; 3 COMSATS University Islamabad, Abbottabad Campus, Abbottabad, Pakistan; 4 Department of Civil Engineering, Nazarbayev University, Astana, Kazakhstan; 5 Operation and Maintenance, Operation, Maintenance and Acoustics, Department of Civil, Environmental and Natural Resources Engineering, Lulea University of Technology, Luleå, Sweden; 6 Department of Civil Engineering, College of Engineering in Al-Kharj, Prince Sattam Bin Abdulaziz University, 11942 Al-Kharj, Saudi Arabia; 7 Department of Transport Systems, Traffic Engineering and Logistics, Faculty of Transport and Aviation Engineering, Silesian University of Technology, Krasińskiego 8 Street, 40-019 Katowice, Poland; 8 National Institute of Transportation, National University of Sciences and Technology (NUST), Islamabad, Pakistan

**Keywords:** TiO_2_, Photocatalytic degradation, Air contaminants, Machine learning, Civil engineering, Environmental impact

## Abstract

The escalation of global urbanization and industrial expansion has resulted in an increase in the emission of harmful substances into the atmosphere. Evaluating the effectiveness of titanium dioxide (TiO_2_) in photocatalytic degradation through traditional methods is resource-intensive and complex due to the detailed photocatalyst structures and the wide range of contaminants. Therefore in this study, recent advancements in machine learning (ML) are used to offer data-driven approach using thirteen machine learning techniques namely XG Boost (XGB), decision tree (DT), lasso Regression (LR2), support vector regression (SVR), adaBoost (AB), voting Regressor (VR), CatBoost (CB), K-Nearest Neighbors (KNN), gradient boost (GB), random Forest (RF), artificial neural network (ANN), ridge regression (RR), linear regression (LR1) to address the problem of estimation of TiO_2_ photocatalytic degradation rate of air contaminants. The models are developed using literature data and different methodical tools are used to evaluate the developed ML models. XGB, DT and LR2 models have high R^2^ values of 0.93, 0.926 and 0.926 in training and 0.936, 0.924 and 0.924 in test phase. While ANN, RR and LR models have lowest R^2^ values of 0.70, 0.56 and 0.40 in training and 0.62, 0.63 and 0.31 in test phase respectively. XGB, DT and LR2 have low MAE and RMSE values of 0.450 min^-1^/cm^2^, 0.494 min^-1^/cm^2^ and 0.49 min^-1^/cm^2^ for RMSE and 0.263 min^-1^/cm^2^, 0.285 min^-1^/cm^2^ and 0.29 min^-1^/cm^2^ for MAE in test stage. XGB, DT, and LR2 have 93% percent errors within 20% error range in training phase. XGB has 92% and DT, and LR2 have 94% errors with 20% range in test phase. XGB, DT, LR2 models remained the highest performing models and XGB is the most robust and effective in predictions. Feature importances reveal the role of input parameters in prediction made by developed ML models. Dosage, humidity, UV light intensity remain important experimental factors. This study will impact positively in providing efficient models to estimate photocatalytic degradation rate of air contaminants using TiO_2_.

## Introduction

**Figure 1:**
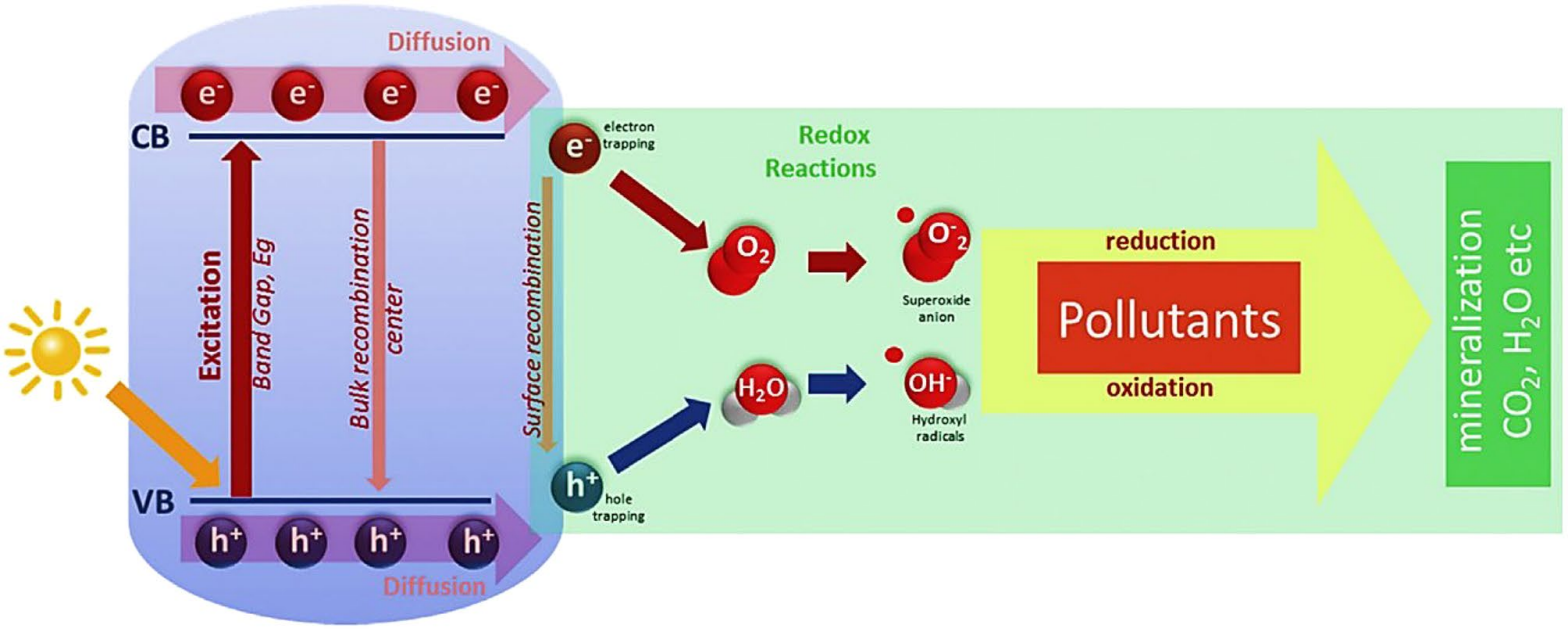
Mechanism of photocatalytic degradation^9^*.*

The escalation of global urbanization and industrial expansion has resulted in an increase in the emission of harmful substances into the atmosphere and waterways. These emissions had detrimental effects including the spread of diseases, aggravation of global warming, and occurrence of unusual climatic conditions. Indoor air pollution has been linked to a condition known as "sick home syndrome"^1^. Furthermore, the release of air pollutants plays a significant role in the creation of environmental threats such as stratospheric ozone depletion, urban smog, and the greenhouse effect. Airborne contaminants provide a significant risk to human health and the environment, necessitating the development of innovative strategies for mitigating their effects. Titanium dioxide (TiO_2_) is a popular choice for pollutant degradation due to its ability in heterogeneous photocatalytic degradation^2–4^. It is very effective at breaking down pollutants with a variety of functional groups and structures due to its non-selective photocatalytic characteristics. Previous research has demonstrated that TiO_2_ has shown encouraging results in the removal of air and water contaminants^5^. TiO_2_ offers several advantages, including cost-effectiveness, low energy consumption, exceptional efficiency, strong oxidizing capabilities, chemical stability, resistance to acids, ease of production, and insolubility in water. The results demonstrate the effectiveness of TiO_2_ in eliminating pollutants, and the photocatalytic process can be carried out at standard ambient temperature and pressure conditions^1,6–8^. Figure 1 illustrates the process of semiconductor photocatalytic degradation.

Moreover, TiO_2_ finds application in a wide range of areas, including air purification, water treatment, water splitting, renewable energy production, and the conversion of carbon dioxide into hydrocarbons^10–14^. In recent decades, extensive efforts have been dedicated to the development of photocatalysts and the assessment of their effectiveness in municipal water treatment processes^15–19^. Nevertheless, quantifying the efficiency of photocatalysts across a spectrum of contaminants presents a difficult challenge. The photocatalytic degradation performance of contaminants is intricately linked to the characteristics of photocatalysts, encompassing factors such as crystalline structure, grain size and shape, specific surface area, pore structure, and more^20–23^. Additionally, a number of experimental factors, including as the dosage of the photocatalyst, the medium's pH, the concentration of contaminants, the wavelength and intensity of the light, and others, have an important impact on the photocatalytic activity^24–26^. The conventional experimental methods for assessment of photocatalytic activity are UV–Vis spectroscopy^27–29^, gas chromatography analysis^30,31^, chemical oxygen demand^29,32^, fluorescence spectroscopy^33^, electrochemical methods^34^. These methods of experimentation require longer reaction times to get measurable contaminant degradation, which causes delays in the collection of results. Moreover, these experimental techniques need complex sample preparation procedures and manual data processing, which requires skilled workers and significant time commitments. The utilization of traditional methods frequently provides limited mechanistic understanding of the photocatalytic degradation process, creating difficulties in the catalyst design and optimization of reaction condition. Experimental processes are important to get quality data for training and testing of ML models but performing repeated experiments for each project are not feasible. An effective way to get over these restrictions and improve estimation of photocatalytic activity is using machine learning models. ML models are capable of accurately and quickly predicting experimentation results and reaction kinetics outperforming more conventional approaches therefore artificial intelligence and ML modelling have been used extensively^35–39^. This facilitates a quicker evaluation of photocatalytic performance. ML algorithms can be highly effective in identifying the ideal reaction conditions to maximize photocatalytic activity and selectivity by utilizing datasets of experimental parameters and reaction results. More sophisticated algorithms are excellent at deciphering complex correlations between catalyst characteristics, reaction parameters, and photocatalytic efficiency, providing crucial mechanistic insights into the degradation process.

Data-driven machine learning offers a novel method for assessing photocatalyst performance that is quick, affordable, and flexible outperforming traditional testing. Artificial neural networks (ANNs) is an established machine learning technique used to forecast properties of many different types of materials, including metals, polymers, composites, and ceramics^40–46^. ANNs have been used to forecast the photocatalytic activity of novel catalysts^47–51^ and to speed up the design and discovery of new catalysts^52–54^. Prior research has employed the gaussian process regression model to forecast the band gaps of anatase TiO_2_ photocatalysts by considering their surface area and lattice properties^55^. It is important to note that these model predictions may not fully account for all the factors influencing photocatalytic degradation, which could leave gaps in our understanding of TiO_2_'s overall performance. Furthermore, it is crucial to conduct a comprehensive comparison to determine which model performs optimally for a given dataset. One of the many crucial steps in developing a successful ML model is evaluating and contrasting many models in order to choose the optimal one for deployment^56^. Existing research indicates that there is no universally superior method, as no single approach consistently outperforms others^57,58^. Many studies tend to introduce new models and only compare them with similar ones, making it challenging to assess their relative and overall performance^59^. Although certain methods may exhibit superior average performance, there exists substantial variability when considering different problems and metrics^58^. Hence, the comparative analysis of diverse algorithms becomes indispensable in the quest to pinpoint the most appropriate model for a particular problem. Distinct models demonstrate excellence under varying circumstances and the utilization of a spectrum of models enhances the effectiveness of research endeavors. Through the comparison of multiple algorithms, researchers can mitigate potential biases and gain a more comprehensive understanding of the problem landscape, revealing patterns and correlations within the data.

In this study, thirteen machine learning techniques are employed to develop machine learning models for prediction of TiO_2_ photocatalytic degradation of air contaminants. The algorithms encompassed linear approaches (such as linear, ridge, lasso, and support vector regression), decision trees, random forests, K-nearest neighbors, and boosting-based methods. The crucial issues of hyperparameter optimization and overfitting are addressed using grid search technique and K-fold cross validation. The dataset for ML models training and testing is acquired from literature and various methods including regression analysis, statistical metrices and visualization techniques are used to evaluate and compare performance of proposed models. Furthermore, feature importances are used to analyze the role of input parameters in predicting the output. This study will improve the prediction process for TiO_2_ photocatalytic rate and knowledge and functionality of different constituents in photocatalytic degradation.

## Overview of employed ML techniques

Thirteen machine learning techniques were assessed to identify the ideal model that can be used to predict TiO_2_ photocatalytic degradation of air contaminants. The algorithms used in this study are discussed in the following sections. Detailed description of employed ML techniques is not scope of this study and can be found extensively in literature^60–64^.

### XG boost (XGB)

**Figure 2:**
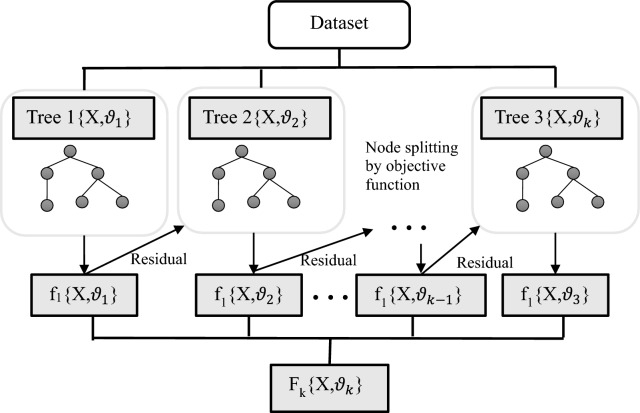
XGB mechanism.

XGBoost is an open-source machine-learning library introduced by Chen and Guestrin in 2016^65^. It stands out as a powerful, versatile, and portable tool. It excels in addressing regression, classification, and ranking problems within supervised learning. Data scientists and machine learning experts widely regard XGB as a dependable and efficient algorithm, particularly when dealing with extensive datasets rich in features. The core principle of XGB involves constructing decision trees sequentially. What sets it apart is its ability to assign greater importance to the factors that previous decision trees may have miscalculated. By combining these numerous classifiers and predictors, XGB produces robust and dependable models. Figure 2 represents the mechanism of XGB. XGB's sequential ensemble learning is illustrated by building decision trees iteratively and training each one to correct the residuals of the previous trees to produce a strong prediction model.

### Decision tree (DT)

**Figure 3:**
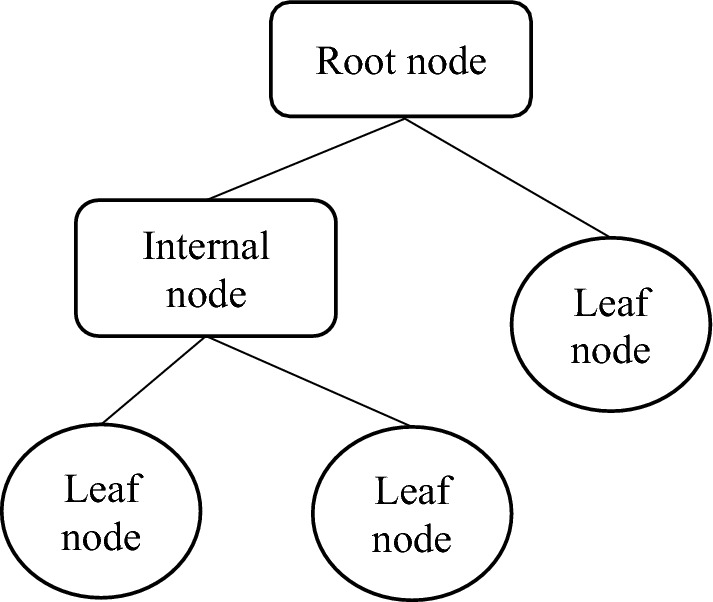
Decision Tree typical three-layer construction.

A decision tree analyzes the data to identify patterns and establish predictive rules. Figure 3 illustrates a three-layer construction of decision tree. The primary objective of a regression tree is to divide the predictors into segments, enabling the prediction of target variables based on these partitions of input variables. Furthermore, the regression tree implicitly selects variables and highlight variables with greater significance in predicting target variables based on the previous nodes in the tree. One of its notable advantages is its versatility in handling both numeric and categorical data. As a result, this approach is considered relatively straightforward, although it requires careful consideration to prevent data overfitting. The disadvantage of regression trees is that their models are inherently unstable. Slight modifications to the dataset might result in completely different partitions, making the optimal model selection difficult^66,67^. Consequently, decision tree models are susceptible to overfitting. To mitigate this issue, more complex tree-based methods like random forests and boosted trees are often preferred for their greater reliability.

### Lasso regression (LR2)

Lasso regression (LR2) is employed for feature selection and regularization to enhance model accuracy^68^. Similar to ridge regression (RR), LR2 minimizes the residual sum of squares by considering the regression coefficients as the sum of absolute values. As shown in Eq. (1), the objective is to minimize the following expression:

1 $${\widehat{\beta }}_{lasso} = {argmin}_{\beta } \left[\sum_{i=1}^{N}{\left({y}_{i} - {\beta }_{0} - {\sum }_{j=1}^{m}{X}_{ij}{\beta }_{j}\right)}^{2} + \lambda {\sum }_{j=1}^{m}\left|{\beta }_{j}\right|\right]$$

**Figure 4:**
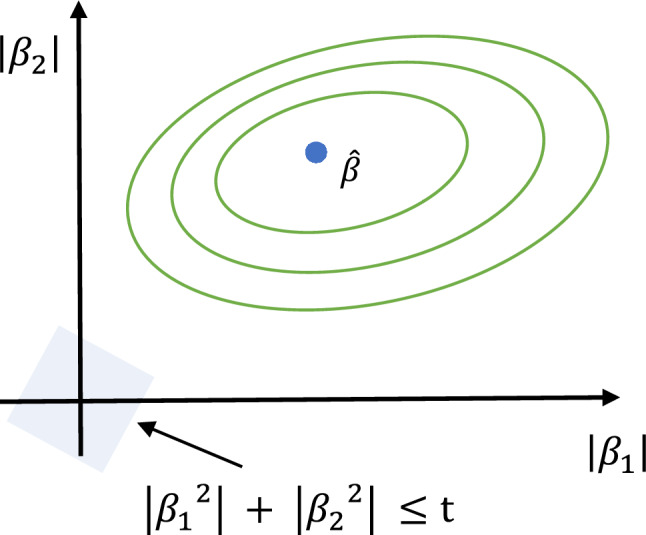
Illustration of lasso regression.

Here, sample numbers in the dataset are indicated by i, while the number of input characteristics is shown by m. The shrinkage parameter is denoted by λ, and as λ grows, the coefficients (βj) tend to approach zero. Figure 4 Illustrates the shrinkage effect and sparsity induction in feature selection using the L1 constraint of Lasso regression on model coefficients. The regularization and the cost function contour intersect to get the ideal coefficients.

### Support vector regression (SVR)

**Figure 5:**
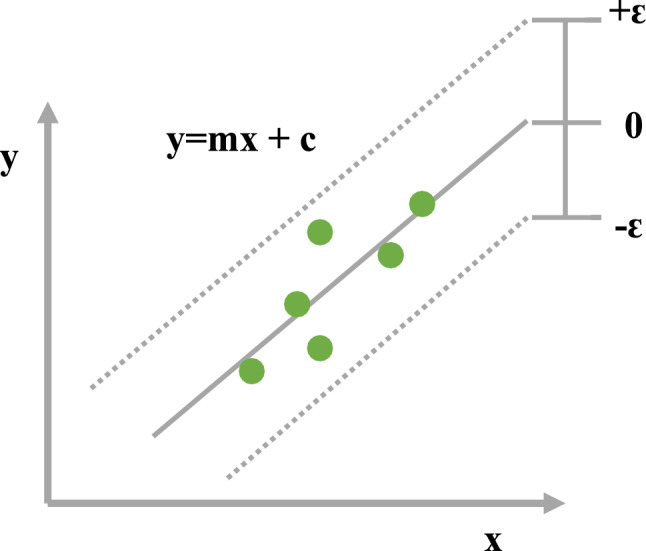
Illustration of KNN process.

Support vector machines (SVMs) are commonly used for support vector regression (SVR), which was introduced by Vapnik et al.^69^. To reduce generalization error, the SVR technique builds a model based on the training data and uses a hyperplane that maximizes the distance between labeled classes to categorize new data points. The sum of the distances from the hyperplane to the closest labeled data points is used to calculate the margin in SVR. SVR minimizes both observable training errors and generalized errors to achieve generalized performance. The SV method is illustrated in Fig. 5, where 'x' stands for the input vector, 'y' for the target value, and the model utilizes the linear regression function.

### AdaBoost (AB)

**Figure 6:**
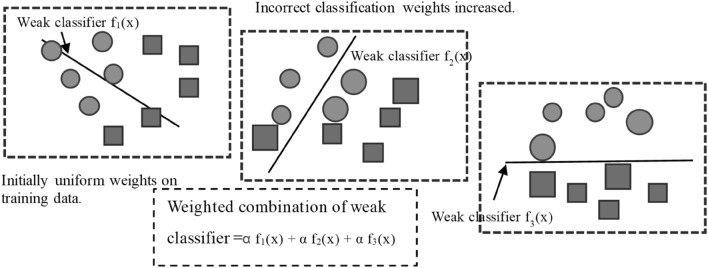
AdaBoost process.

Boosting is an ensemble technique that combines multiple weak learners to create a strong learner. It involves adjusting the sample weights of the training data for each model based on the results of the previous model. This means that the outcomes of previous learning iterations influence subsequent ones, leading to an increase in the importance of data over boosting rounds. AdaBoost, a specific boosting algorithm, focuses on increasing the weight of training samples that were poorly predicted by the previous model. Initially, AdaBoost creates the first weak learner and assigns output weight (C) and model weight (w) to each data point based on the learning results. The model is then updated with the data weights. This process is repeated for a specified number of iterations (N). Data points that were incorrectly predicted by the previous models are given higher weight, while those accurately predicted have their weight reduced. As the iterations continue, challenging examples receive increasing attention. After N iterations, each of the N weak learners is assigned a model weight (w) to contribute to the final model^66,70^. The boosting procedure is shown in Fig. 6, wherein AdaBoost increases the weight of training data that the prior model underfits. The weighted sum of this combination is the result of the AdaBoost model.

### Voting regression (VR)

**Figure 7:**
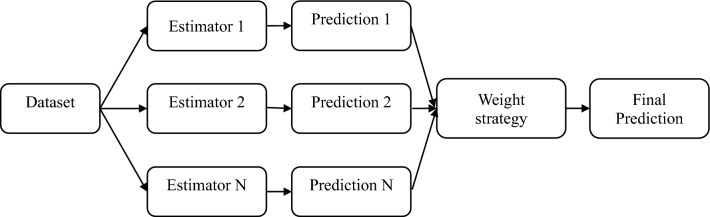
Mechanism of voting regressor.

A voting regressor falls under the category of ensemble meta-estimators, sequentially fits multiple base regressors to the entire dataset^71^. It generates the final prediction by averaging the outputs of these individual estimates. The key advantage of using a voting regressor is its resilience to significant errors or mispredictions from any single model. Since it relies on the collective performance of multiple models, the negative impact of one model's poor performance is mitigated by the strong performance of others. By combining several models, the chances of a single model making an incorrect prediction are minimized. This approach enhances the robustness of the estimator and reduces the risk of overfitting. The voting regressor workflow illustrated in Fig. 7 shows that many estimators work in parallel to provide predictions, which are then aggregated using a weighted technique to get a final prediction that is less likely to be overfit and more reliable.

### CatBoost (CB)

**Figure 8:**
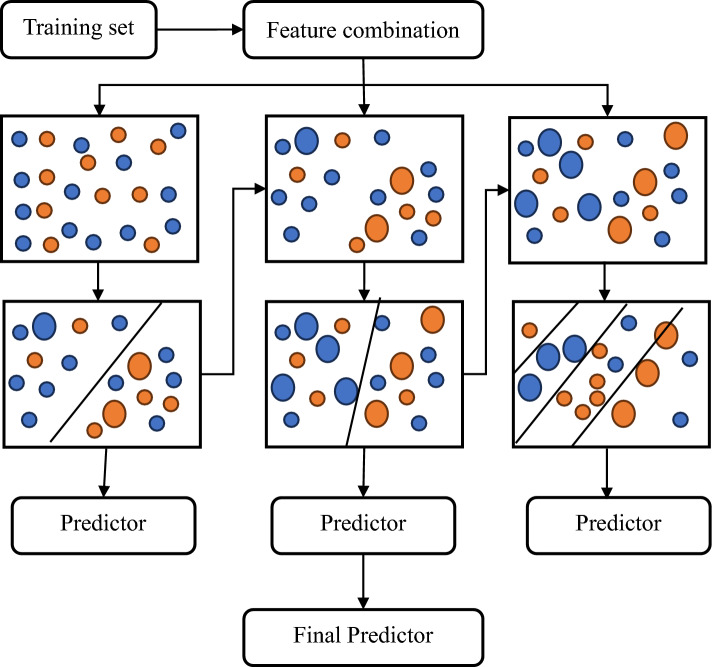
Schematic diagram of CatBoost regressor.

CatBoost (categorical boosting) is an open-source gradient-boosting method for decision trees that was first presented by Prokhorenkova et al. in 2018^72^. Its unique, efficient, and greedy gradient-boosting methodology make it distinctive. CatBoost is utilized in a variety of domains, including recommendation systems, ranking, prediction, and even personal assistants, in addition to regression and classification challenges. One of its notable advantages is that it may reduce the need for extensive hyperparameter adjustments, which minimizes the risk of overfitting, thus giving models more applicability. Due to its use of symmetric decision trees, this method can rapidly infer pre-trained weak learning models. It is particularly well-suited for handling noisy data with diverse attributes and complex relationships. Figure 8 is schematic of the CatBoost regressor illustrating the training of several predictors using different feature combinations from the training set, with an ensemble of the judgments made by each predictor serving as the final prediction.

### K-nearest neighbors (KNN)

**Figure 9:**
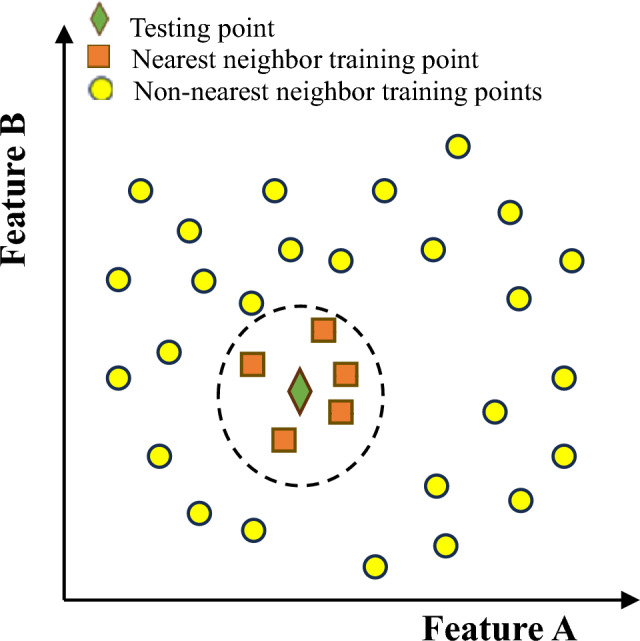
Illustration of KNN.

The K nearest neighbor (KNN) technique involves making predictions for new records by comparing them to the most similar records in a dataset. Figure 9 provides a visual representation of KNN algorithms. It can be applied to both regression and classification tasks in machine learning. KNN operates on the assumption that observations close in the attribute space (e.g., concrete mix properties) are also close to each other in terms of output values. Predictions for output values are made by using a predefined function of the response values of the nearest neighbors, with a focus on the closest neighbors in the data space. In standard KNN, the average function is commonly used. Some key properties of standard KNN include: 1. Assigning equal importance to all neighbors and using the average function to calculate the response value for unknown observations. 2. Treating all normalized attributes as equally important by assigning the same weight to them. 3. Using Euclidean distances to calculate distances between data points. An advantage of KNN is its resilience to noise in the training data, making it an effective algorithm for handling large datasets^66,67^.

### Gradient boost (GB)

**Figure 10:**
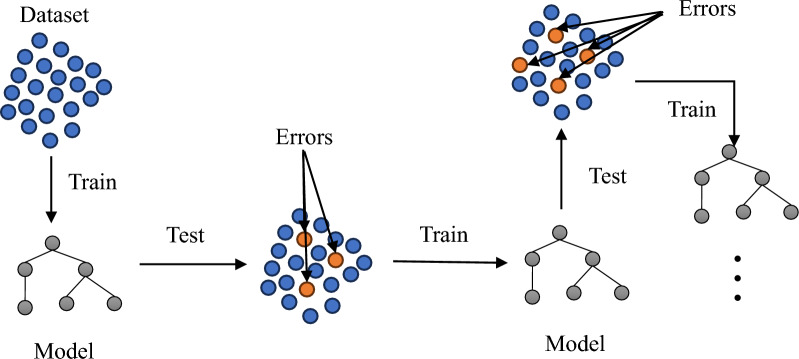
Process of gradient boosting.

Gradient boost (GB) is a machine learning boosting strategy that uses decision trees as weak learners in order to minimize the total error of the model and produce a robust learner^61^. This strategy employs an iterative ensemble methodology in which a weak learner is introduced and trained by the algorithm to minimize the overall training error. Then, until the model's overall error hits the target level, another weak learner is added and trained similarly. The basic method of GB is to perform regression on a function that is obtained from the gradient vector of the loss function that was computed in the preceding iteration^73^. Figure 10 illustrates gradient boosting mechanism in which decision trees are iteratively trained on progressively larger residual errors until an improved ensemble model is produced.

### Random forest (RF)

**Figure 11:**
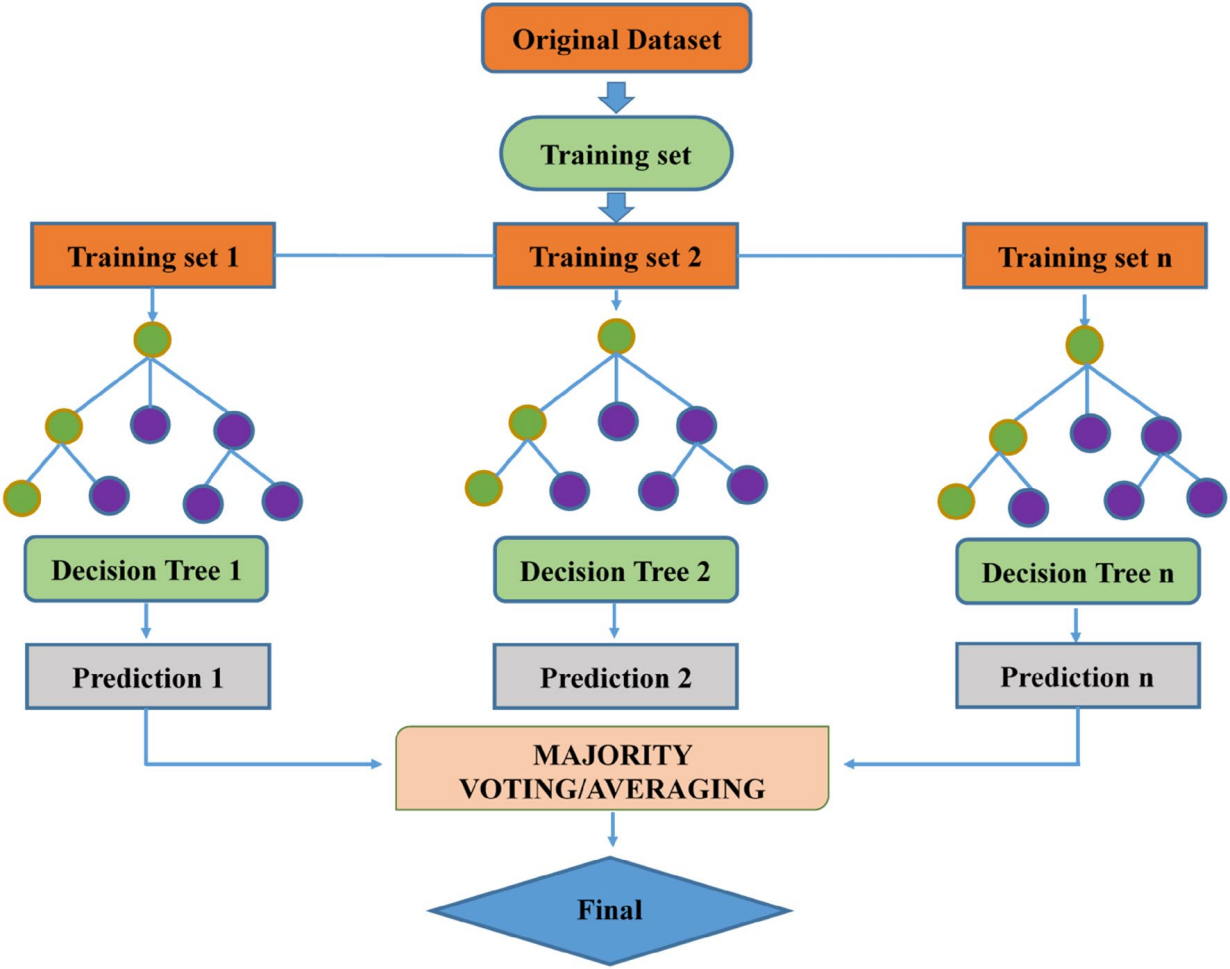
RFR mechanism^63^*.*

Random forest involves constructing an ensemble of regression trees to mitigate the variability observed in individual trees. In this approach, decision trees come together to form a "forest" by applying the concept of "bootstrap aggregation" (bagging), which entails creating multiple similar datasets sampled from the same source dataset. Bagging involves combining trained base models on different subsets of the training data. Decision trees are known for their low bias but high variance, making them susceptible to overfitting. The key advantage of the random forest method lies in its ability to significantly reduce this instability. However, decision trees, when used in isolation, tend to overfit the training data. To address this issue and prevent overfitting, random forest models are typically constructed by aggregating multiple decision trees or through regularization techniques^66,74^. An illustration of the RF process is shown in Fig. 11. First, the size of the samples is established, and then each sample has a decision tree constructed for it. Based on the input parameters entered, each decision tree generates a prediction. The best forecast is determined by a voting procedure in which the result receiving the most votes is declared the best prediction. The majority vote ultimately determines the outcome of the final forecast.

### Artificial neural network (ANN)

**Figure 12:**
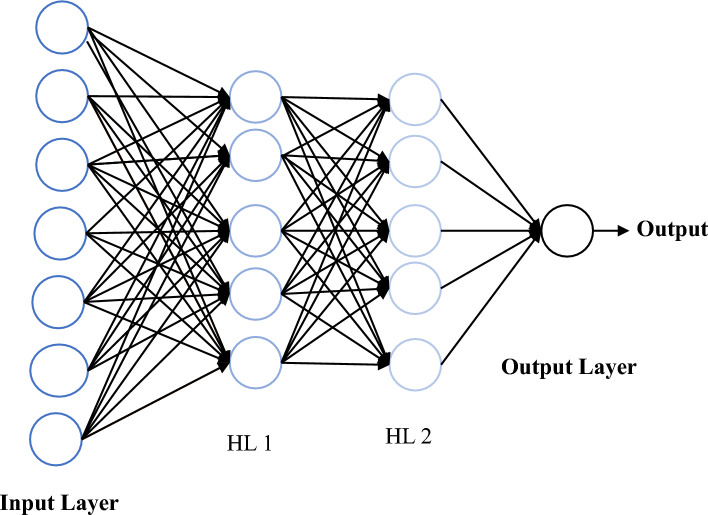
Mechanism of artificial neural network with 2 hidden layers.

The artificial neural network (ANN) is an algorithm rooted in deep learning, aiming to emulate the human brain and its neural system^75^. It comprises neurons as processing elements that form interconnected networks, with each processing element having multiple inputs and generating a single output. When the input to a processing element surpasses a certain threshold, it transmits information to neighboring elements. The strength of these connections between processing elements is determined by assigning weights, which are adjusted during the training process using training data to minimize the difference between predicted and target values. The hidden layer, situated between the input and output layers, plays a crucial role by applying nonlinear transformations to the inputs. An ANN's operation is shown in Fig. 12. The hidden layers, which include algorithms that replicate neural processes in the human brain, receive data from the input nodes. The network learns from the input nodes, which leads to activation inside the hidden nodes. The output layer generates predictions because of the patterns and insights that these hidden layers have extracted from the data.

### Ridge regression (RR)

Ridge regression (RR) shares similarities with LR1 but focuses on reducing the model variance observed in LR1^68^. In RR analysis, the coefficients are shrunk to minimize the residual sum of squares, as illustrated in Eq. (2).

2 $${\widehat{\beta }}_{ridge} = {argmin}_{\beta } \left[\sum_{i=1}^{N}\left({y}_{i} -{B}_{0} - {\sum }_{j=1}^{m}{X}_{ij} {B}_{j}\right) + \lambda {\sum }_{j=1}^{m}{{\beta }_{j}}^{2}\right]$$

**Figure 13:**
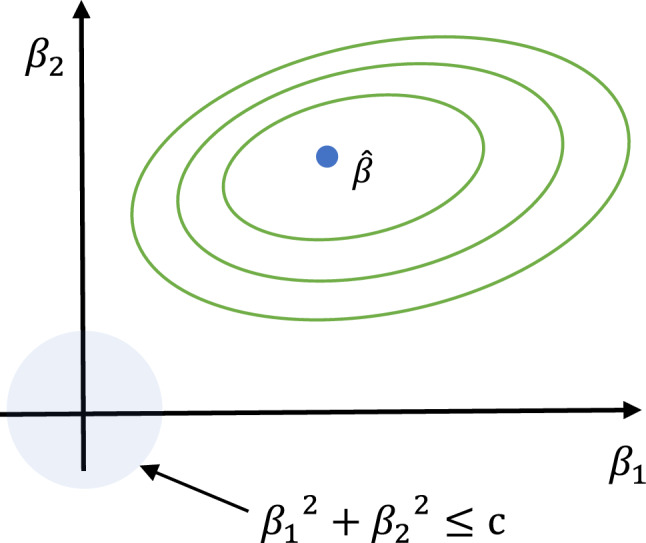
Process of ridge regression.

The notations are the same as Eq. (1) in Lasso regression. Here, m represents the number of input features, and i represents the sample numbers in the dataset. The shrinkage parameter is denoted as λ, and as λ increases, the coefficients (βj) tend to approach zero. Ridge regression illustrated graphically in Fig. 13, showing the restriction placed on coefficients (β) by L2 regularization inside a circle, which lowers variance and strengthens the model's resistance to multicollinearity.

### Linear regression (LR1)

**Figure 14:**
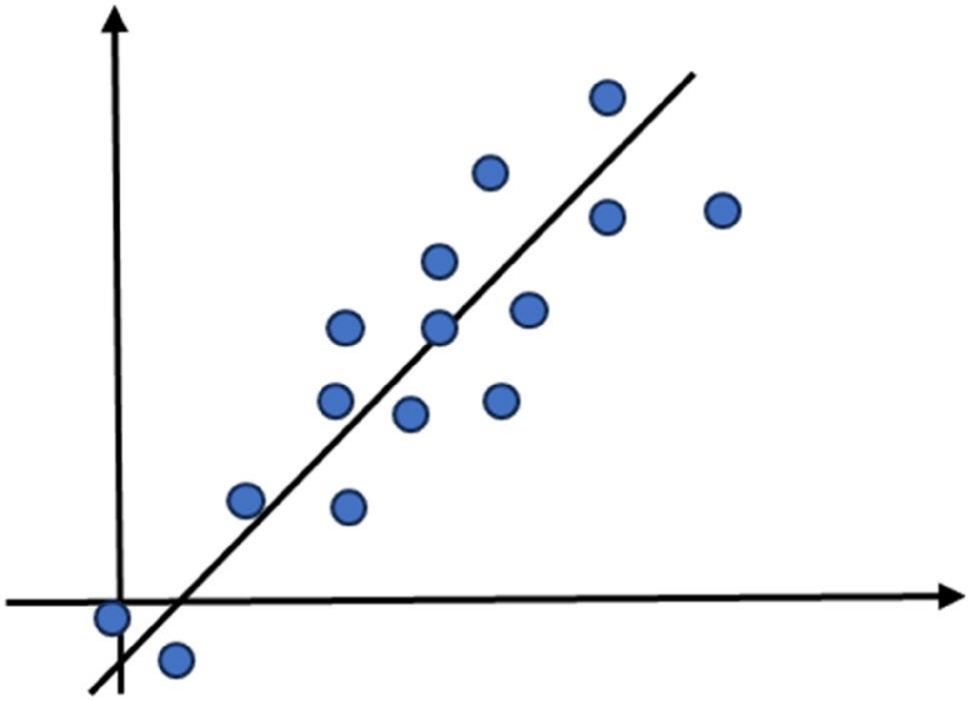
Process of Linear regression.

An established linear connection between dependent and independent variables is established by the supervised machine learning approach known as linear regression (LR1)^76^. This technique uses gradient descent to get the best coefficients and gives regression coefficients to explain this linear connection. LR1 models the target variable's prediction value based on the supplied input variables, also known as independent variables. In the m-dimensional space, where m is the number of independent characteristics, it fits a hyperplane. The regression coefficients are chosen such that the cost function is minimized. After this minimization procedure, the best-fit line is obtained using gradient descent, where the ideal set of regression coefficients is selected. The process begins with random values for θi and iteratively updates them to reduce the cost function value.

3 $$Y= {\beta }_{0} + \sum_{j=1}^{m}{X}_{j}{B}_{j}$$

where (Y) is target variable and Xj is independent variable and βj is regression coefficients. Figure 14 shows a scatter plot that has a linear regression line to illustrate the idea of fitting a linear model to the data points can be used to highlight the predicted connection between an independent and dependent variable.

## Methodology

### Source and features of literature data

The database utilized for ML model development contains 200 sets of experimental data derived from previous research^77^. The dataset includes seven independent variables, which essentially cover the majority of significant experimental condition settings: dosage, ultraviolet light intensity, humidity, wavelength, experimental temperature, initial concentration of air contaminant and reactor volume. The output variable for the ML models was photocatalytic degradation rate of TiO_2_ (k, min^-1^/cm^2^). The reaction rate absolute values are usually very small positive number therefore they are converted to base 10 logarithm -log(k). Table 1 shows the statistical analysis of dataset. The mean, minimum (min), and maximum (max) indicate the center and extreme data points. Standard deviation (SD) is used to measure the dispersion of the data. A smaller SD suggests that most data points are close to the mean, while a larger SD indicates greater dispersion across the range of values. Skewness and kurtosis are employed to assess the symmetry and shape of the data distribution, relative to a normal probability distribution. Both can be zero, positive or negative and maybe undefined^78^. The data curve is flatter than the normal distribution curve when kurtosis is negative and more peaked than when it is positive. Conversely, the data distribution is normal with a medium peak on zero kurtosis value^79–81^. A skewness value of exactly zero indicates asymmetry, whereas positive and negative values indicate that the probability distribution curve's tail is stretched to the right and left of the curve, respectively^82^. Generally, acceptable ranges for kurtosis and skewness and kurtosis are between − 10 to + 10 and − 3 to + 3 respectively^79,80,82,83^.

**Table 1:** Statistical analysis of dataset.

Input variables	Notations	Mean	Min	Max	Std	Kurtosis	Skewness
Dosage (mg/cm^2^)	D	2.84	0.01	51.40	6.37	31.81	5.36
Humidity (%)	H	48.53	0.00	300.00	35.23	12.26	2.24
Ultraviolet light intensity (mW/cm^2^)	I	12.23	0.36	30.00	11.60	− 1.45	0.53
Initial concentration of air contaminant (ppmv)	Initial C	165.23	0.00	1943.00	311.93	11.81	3.17
Reactor volume (L)	R	1.79	0.04	6.30	1.98	− 0.44	1.02
Experimental temperature (°C)	T	39.64	22.00	97.50	19.31	0.42	1.22
Wavelength (nm)	W	321.32	250.00	370.00	48.52	− 1.52	− 0.53
Output variable							
Photo-degradation rate (min^-1^/cm^2^)	log(k)	3.29	0.00	9.80	1.39	7.88	2.20

### Data preprocessing

Data preprocessing transforms raw, unclean data from various sources into clean and analyzable data. Raw data often come in formats that complicate or outright prevent analysis and machine learning application. For machine learning projects, it's crucial to format the data correctly to enhance the performance of the chosen models. Each MLmodel has its specific requirements for data format. Organizing the dataset appropriately is key to effectively applying and comparing different machine learning and deep learning algorithms. This includes removing missing values, converting categorical data into numerical values, which is vital for many of these algorithms. Moreover, dataset standardization is a common prerequisite involving scaling numerical data to fit within a certain range, removing the mean and adjusting to unit variance to ensure consistency and improve algorithm efficiency. In this study, data preprocessing and visualization is performed using python on Jupyter notebook environment. The StandardScaler tool of scikit-learn is used for data standardization. The formula for standardization is given in Eq. 4.

4 $$z=\frac{\left(x- \mu \right)}{\sigma }$$

where “z” is standardized value, and “x” were original value of feature. "μ" and "σ" is the mean and standard deviation of the feature values. After removing anomalies from dataset, correlation and parametric studies were performed to assess the features involved in the data.

#### Correlation study

**Figure 15:**
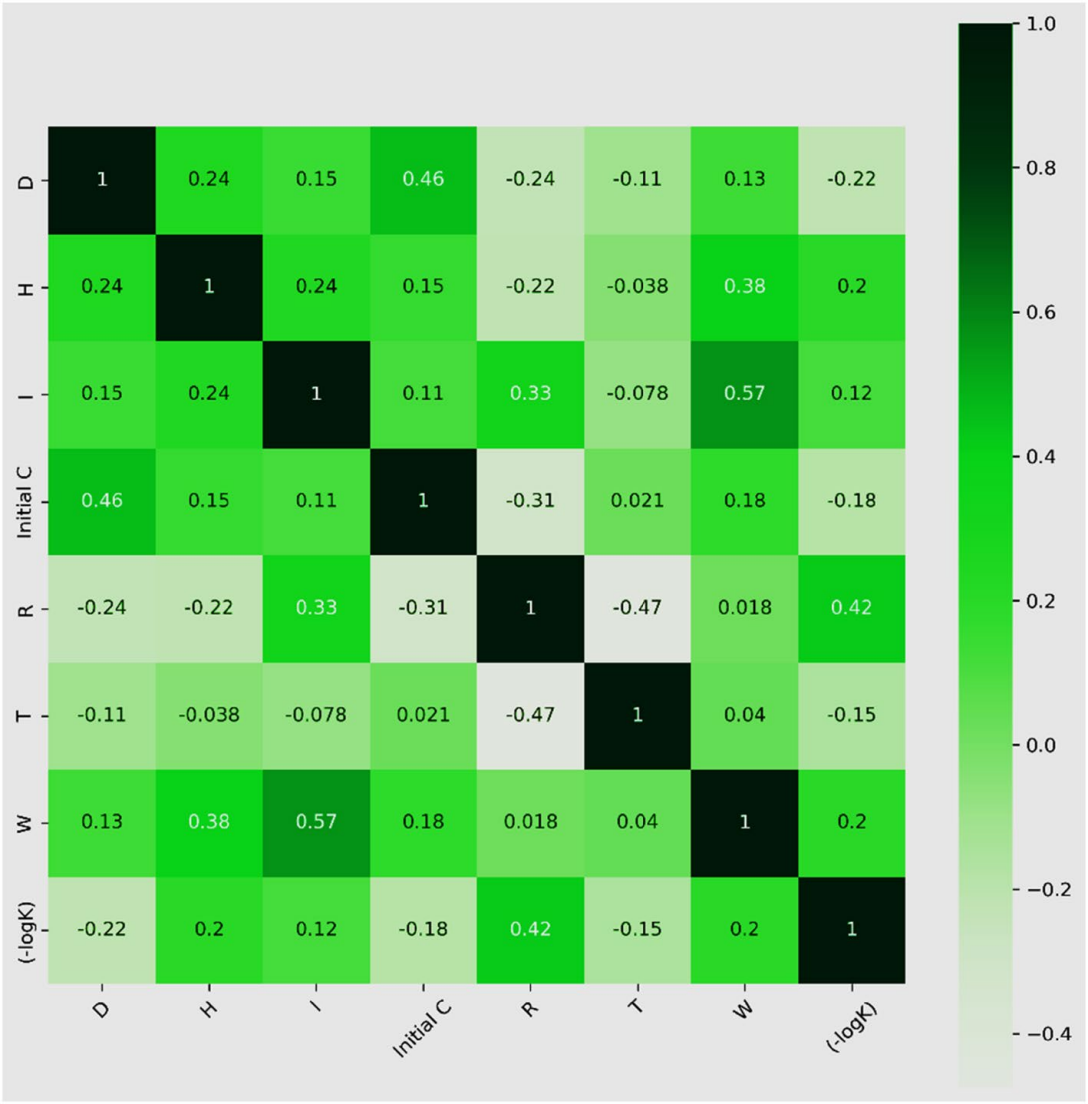
Correlation matrix of dataset.

The correlation matrix of the input data is shown in Fig. 15. Lighter hues signify weaker correlations, whereas darker hues suggest greater positive correlation. Notably, there is a large negative correlation (R = − 0.47) between T and R and a high positive correlation (R = 0.57) between variables W and I. High correlation values (|r|> 0.5) among the input variables enhance the possibility of multicollinearity, which can lead to bias and affect model results. The variance inflation factor (VIF) is an essential diagnostic tool in statistical research that helps identify collinearity between independent variables^84^. VIF is a quantitative indicator of collinearity inside a regression model, and its values normally span from 0 to 10 and are occasionally limited to 0 to 5^85^. Multicollinearity may be evaluated using the tolerance value which is the reciprocal of the VIF. In general, tolerance values between 0.1 and 1 are seen as a sign of negligible multicollinearity^86^. Table 2 shows that there is no substantial multicollinearity among the independent variables in this dataset as shown by tolerance values more than 0.1 and VIF values less than 5^86^.

**Table 2:** VIF analysis of dataset.

		Inputs						
		Dosage	Humidity	I	InitialC	Reactor Volume	Experimental temperature	Wavelength
Collinearity statistics	VIF	1.45	1.52	2.1	1.39	2.62	1.5	1.77
	Tolerance	0.688	0.656	0.498	0.718	0.382	0.67	0.57

#### Parametric study

**Figure 16:**
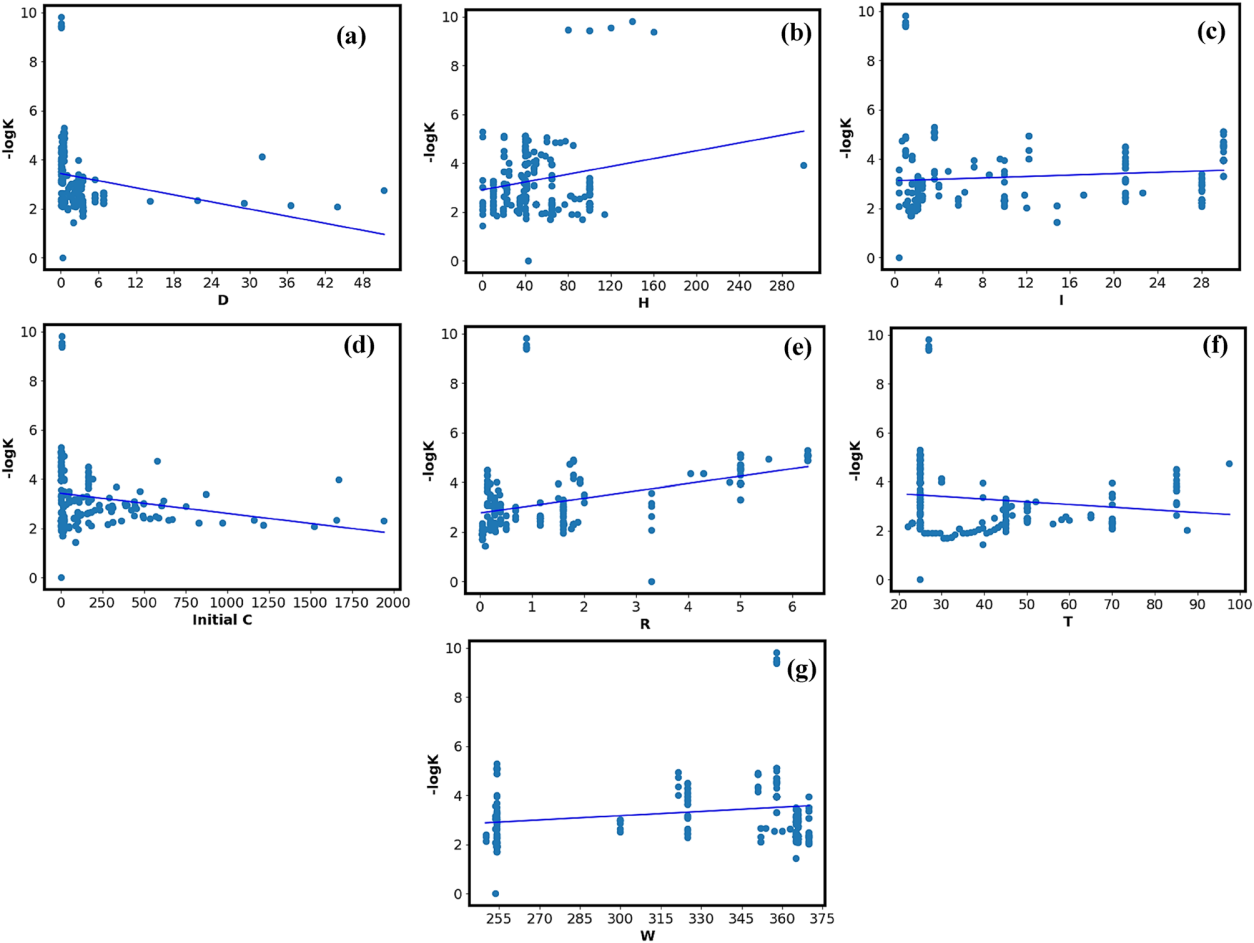
Parametric study of dataset (**a**) dosage (**b**) humidity (**c**) UV intensity (**d**) initial C (**e**) reactor volume (**f**) temperature **g**) wavelength.

Figure 16 shows the relationship between the photocatalytic degradation rate and the dataset's seven input factors. Figure 16(a)–(d) shows that factors such as dosage, UV light intensity, humidity, and InitialC have a significant impact on the photocatalytic degradation rate. A progressive decrease in the rate of photocatalytic degradation rate is evident when D is present. In contrast, Fig. 16(b) and (e) show that factors like humidity and reactor volume have a favorable effect on photocatalytic degradation rate. A completely linear relationship between the input and output variables is improbable as the measured photocatalytic degradation rate for each input variable is displaying a large range.

### ML models development

The database is divided using random test train split to apply ML modeling. The training and test data ratio was 7.5:2.5. The ratio of input variables to data instances has a significant impact on the accuracy and efficiency of the ML models^87,88^. This ratio must be higher than 5 to assess the reliability of data for developing strong correlations among variables^88,89^. The requirement is efficiently satisfied by the ratio of twenty-one for training data and seven for test data in this study. It is crucial to choose the appropriate hyperparameter for each algorithm to improve the performance of all the models. The hyperparameters utilized for each model are listed in Table 3. The hyper parameter mentioned in Table 3 are adjusted using the grid search approach^90^ along with tenfold cross-validation to improve generalizability and robustness of models.

**Table 3:** Hyper-parameters values of the proposed Machine Learning models.

Models	Model parameter	Value
XGB	Subsample ratio for training instances	0.95
	Learning rate	0.05
	Minimum child weight	5
	Column subsampling rate for each tree	0.75
	Estimators max number	200
	Maximum depth of each tree	7
DT	Maximum depth of the decision tree,	12
	Split criterion	poisson
LR2	Regularization parameter (alpha)	0.1
	fit_intercept	TRUE
SVR	Regularization parameter, C,	1000
	Degree of the polynomial kernel function	1
	Kernel type	RBF
	Kernel coefficient, gamma	1
AB	Learning Rate	0.01
	loss function	exponential
	Max estimators number	500
VR	KNeighborsRegressor weight	1
	CatBoostRegressor weight	6
	DecisionTreeRegressor weight	2
CB	Bagging temperature	0.2
	Depth	10
	Learning rate	0.05
	Metric period	75
	Evaluation metric	RMSE
	Number of iterations	700
	One hot Type	Iter
	Od Wait	100
	Random Seed	23
	Subsample	1
KNN	Algorithm	auto
		(cont’d)
	Weight function	distance
	Number of neighbors	9
	Leaf Size	45
	Power parameter, p	1
GB	Learning rate	0.05
	Max estimators	500
	Loss function	Squared error
	Min samples leaf node	2
	Max depth	4
	Max features	auto
RF	Number of trees	164
	Min samples leaf node	1
	Min samples for split	2
	Criterion	absolute error
ANN	Activation function	tanh
	Hidden layer size	100, 100, 100
	Alpha (regularization parameter)	0.05
	Max iteration	10,000
	Learning rate	adaptive
	solver	sgd
RR	alpha	16.84
	solver	saga
	fit_intercept	TRUE
LR1	Normalization	FALSE

### Statistical metrics for ML model evaluation

The performance of ML models is also assessed by statistical metrices. These metrices determine the accuracy level of the models in different aspects. The correlation between experimental and predicted results are measured using the correlation coefficient (R). R above 0.8 is considered a significant and robust correlation between the experimental and model-predicted results^91^. However, R have shown insensitivity towards the division and multiplication of given outcomes^92^. Therefore, R^2^ is used due to its unbiased estimate and enhanced performance. The R^2^ values around one represents that maximum variance among the explanatory factors is captured^93^. Nash–Sutcliffe Efficiency (NSE) above 0.65 is required for a model to show good efficacy^83^. The significance of root mean square error (RMSE) lies in its ability to handle larger error values relative to smaller ones^94^. Nevertheless, RMSE might not be enough to guarantee ideal model performance in some circumstances. As a result, the mean absolute error (MAE) is also calculated. MAE gives greater weight to lower error values, works incredibly well with continuous and smooth data^95^. In conclusion, greater NSE and correlation metrics (R^2^), together with lower error statistical measures (MAE, RMSE), suggest better and enhanced model performance. When a model is overfitted to the training set of data, it can lead to serious issues with machine learning approaches. Consequently, testing error tends to increase while training error continues to decrease^96^. The idea of OF was created as a fitness function in machine learning models to mitigate the impact of overfitting. The RRMSE, R, and relative percentage statistical features that are present in training and testing datasets are considered by the OF parameter, which makes it significant. According to the literature, the most accurate model is the one with the lowest OF value^97,98^. Furthermore, recently a new engineering index, the a20-index, has been proposed^99–101^ for the reliability assessment of the developed ML techniques:

5 $$a20 - index = \frac{m20}{M}$$

where m20 is the number of samples having a ratio of experimental value to projected value between 0.80 and 1.20, and M is the number of dataset samples. It should be noted that the unit value of the a20-index values is anticipated for a flawless prediction model. The suggested a20-index indicates the proportion of samples that meet expected values with a ± 20% variation from experimental values, which has a physical engineering significance. Table 4 provides details of statistical parameters employed.

**Table 4:** Statistical parameters for performance evaluation.

Equation of statistical indicator	Acceptable range
${R}^{2}=1- \frac{\sum_{i=1}^{n}({Y}_{i} - {X}_{i}{)}^{2}}{\sum_{i=1}^{n}({\overline{Y} }_{i} - {X}_{i}{)}^{2}}$	Close to 1
$NSE=1-\frac{{\sum }_{i=1}^{n}{({X}_{i }- {Y}_{i})}^{2}}{{\sum }_{i=1}^{n}{({X}_{i }- \overline{{X }_{i}})}^{2}}$	Higher than 0.65 for very good model
$RMSE=\sqrt{\frac{{\sum }_{i=1}^{n}{({Y}_{i }- {X}_{i})}^{2}}{n}}$	MAE < RMSE
$MAE=\frac{1}{n}{\sum }_{i=1}^{n}{ |X}_{i }- {Y}_{i}|$	Lower values
$VAF=\left(1- \frac{var \left(X-Y\right)}{var \left(X\right)}\right)*100$	Higher values
$OF= (\frac{{n}_{T} - {n}_{V}}{n} ){\rho }_{T} + 2( \frac{{n}_{v}}{n} ) {\rho }_{V} ; T=Training\,data,V=Testing\,data$	For good model (less than 0.2)
$PI or \rho =\frac{RRMSE}{1+ R}$	For good model (less than 0.2)

n = data points, Xi = Experimental data, Yi = predicted data, $\overline{{X }_{i}}$= average experiment values, $\overline{{Y }_{i}}$= average predicted values.

## Result and discussion

### Regression analysis

**Figure 17:**
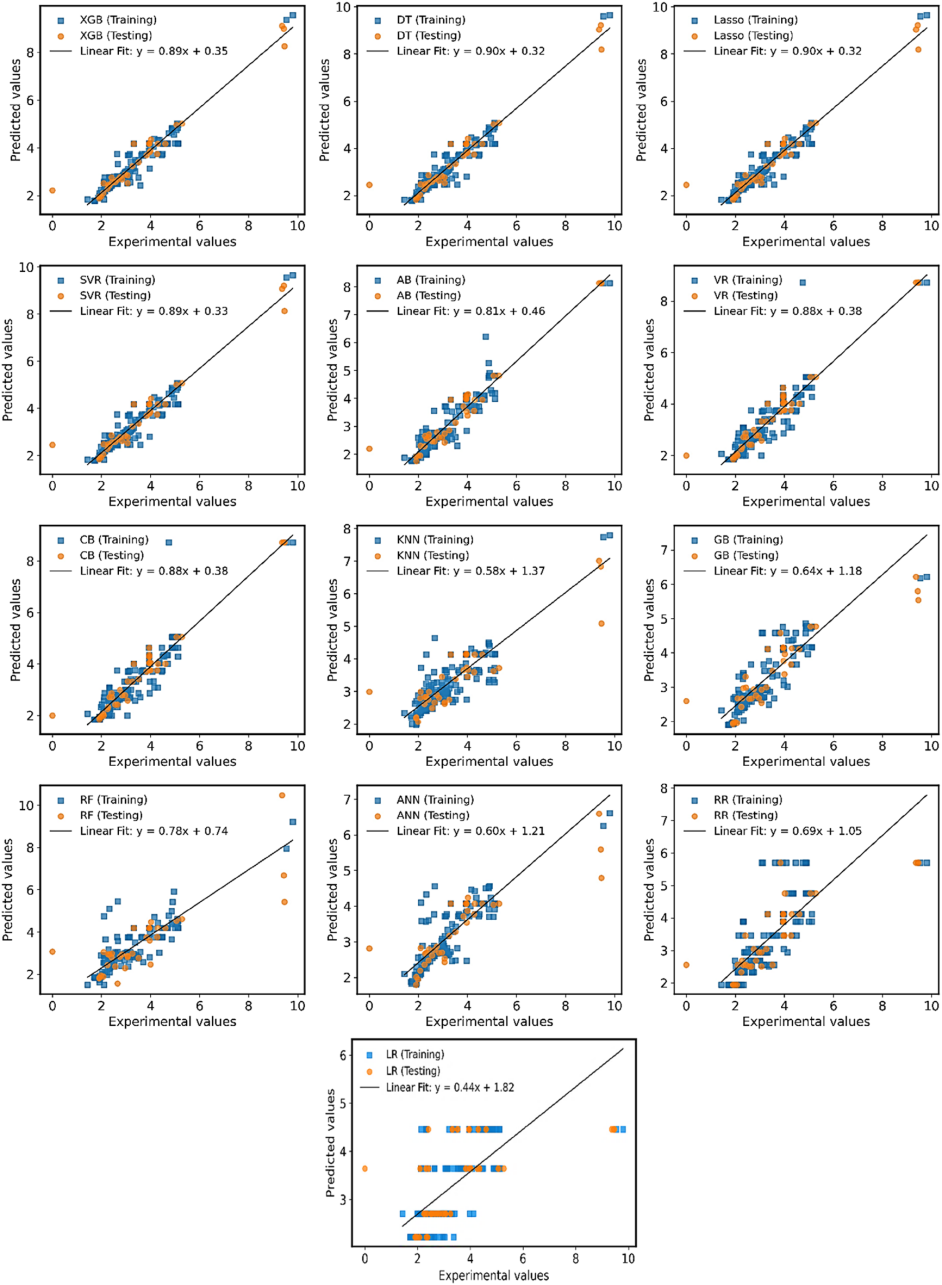
Regression analysis of employed models.

The study employed an optimal mix of hyperparameters to create machine learning models as listed in Table 3**.** A prediction model with a high R^2^ value is typically regarded as superior. Figure 17 represents regression graph for ML models results with x-axis and y-axis representing experimental values and representing predicted outcomes respectively. The linear fitting line represents a good fit between experimental and predicted outcomes. The XGB model demonstrates the highest R^2^ values of 0.932 and 0.937 in the training and test phases, respectively. The DT, LR2, and SVR models also exhibit strong performance, achieving R^2^ values of 0.927 in the training phase and 0.924, 0.924, and 0.923 in the test phase, respectively. Conversely, ANN, RR, and LR1 display lower R^2^ values of 0.62, 0.63, and 0.31 in the training phase, and 0.70, 0.56, and 0.40 in the test phase, respectively. AB, VR, and CB models deliver average performance, with R^2^ values of 0.86, 0.86, and 0.82 in the training phase, and 0.90, 0.93, and 0.93 in the test phase, respectively.

### Statistical analysis of results

**Figure 18:**
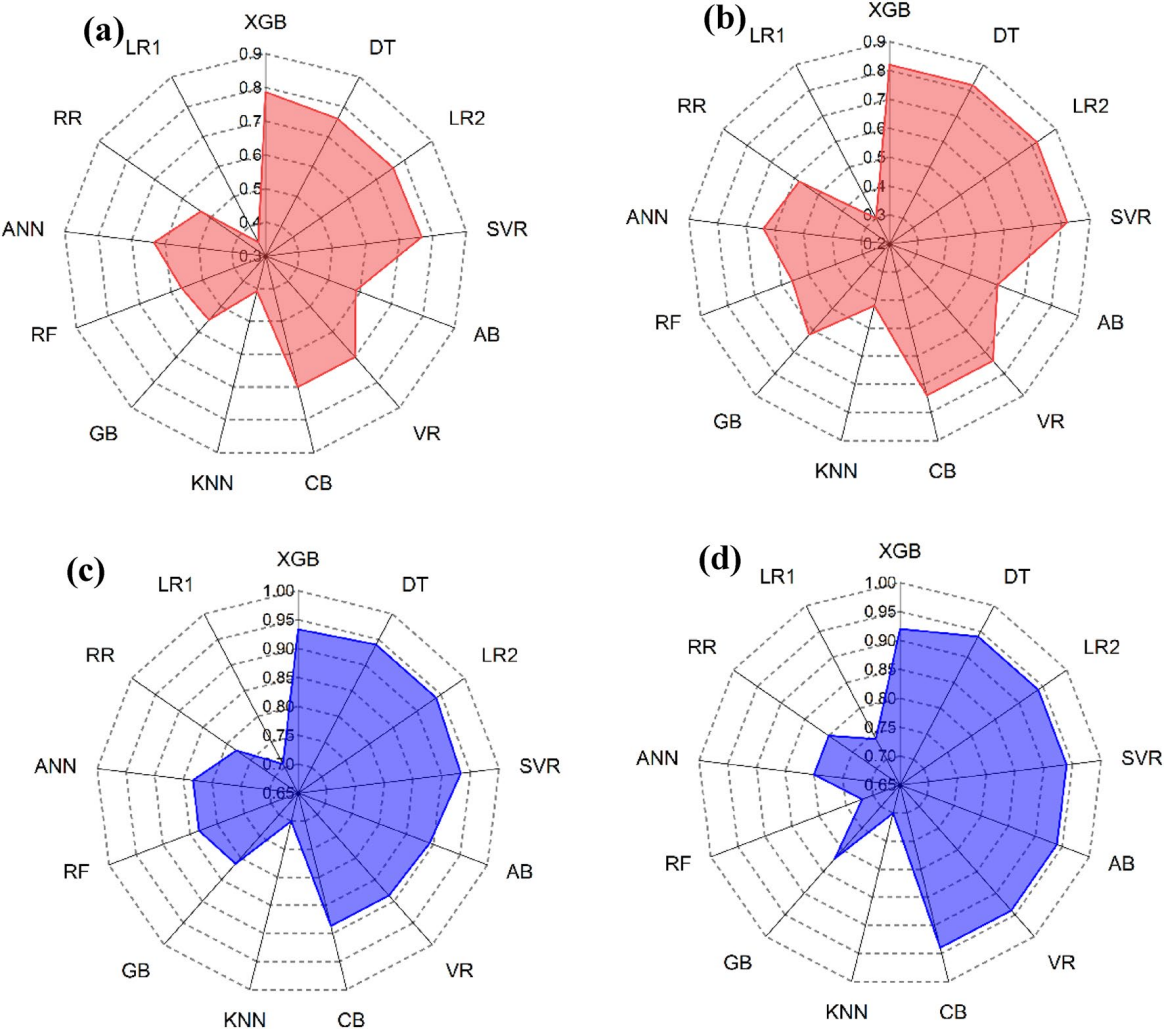
Results of model performance on indicators with physical meaning (**a**) a10 training (**b**) a10 testing (**c**) a20 training (**d**) a20 testing.

The performance of the developed ML models on statistical metrices is shown in Table 5. In the training phase, the XGB algorithm exhibits exceptional performance achieving the best values across multiple performance metrices, including RMSE (0.318 min-^1^/cm^2^), R^2^ (0.932), MAE (0.211 min^-1^/cm^2^). It demonstrates impressive accuracy with an R^2^ of 0.937 in the test phase. XGB technique maintains accuracy in the testing phase with RMSE (0.450 min-^1^/cm^2^), MAE (0.263 min^-1^/cm^2^). Similar, consistent performance is given by DT, LR2 and SVR throughout the training and test phase with lower RMSE and MAE values. On the other end of the spectrum, ridge regression, and linear regression are identified as the worst-performing algorithms. They exhibit lower R^2^ values in both the training and testing phases, indicating their limited accuracy in predicting the target variable. XGB exhibits the most optimal values in terms of PI, VAF%, and OF, with subsequent performance demonstrated by DT, LR2, and SVR. Furthermore, a20 and a10 indices are used which have physical meaning and demonstrate the percentage of prediction with error less than 20% and 10% respectively. Figure 18 shows results for a10 and a20 metrices in train and test phase. XGB has the highest ratio of values within 10 and 20 percent error. Similar performance is shown by DT, LR2, SVR and CB. On other hand, LR2, RF, RR and AB have low values for a10 and a20 indices showing higher errors. XGB, DT, LR2 and SVR have 93% percent errors within 20% error range in training phase. XGB has 92% and DT, LR2 and SVR has 94% errors with 20% range in test phase. Only KNN, RF and LR have more than 70% predictions in the 20% error range while all other models have more than 80% predictions within 20%error range.

**Table 5:** Results of model performance on statistical parameters.

Model/ Statistical	Phase	R^2^	NSE	RMSE (min-^1^/cm^2^)	MAE (min-^1^/cm^2^)	VAF%	PI	a10	a20	OF
XGB	Training	0.932	0.932	0.318	0.211	93.198	0.051	0.787	0.933	
	Testing	0.937	0.980	0.450	0.263	93.738	0.066	0.820	0.920	0.1366
DT	Training	0.927	0.927	0.329	0.228	92.695	0.053	0.760	0.940	
	Testing	0.924	0.983	0.494	0.285	92.436	0.073	0.820	0.940	0.1401
LR2	Training	0.927	0.927	0.329	0.228	92.695	0.053	0.760	0.940	
	Testing	0.924	0.983	0.494	0.285	92.436	0.073	0.820	0.940	0.1401
SVR	Training	0.927	0.927	0.330	0.227	92.661	0.053	0.767	0.933	
	Testing	0.923	0.982	0.496	0.285	92.366	0.073	0.820	0.940	0.1403
AB	Training	0.866	0.866	0.447	0.324	87.926	0.074	0.587	0.893	
	Testing	0.906	0.725	0.550	0.354	91.126	0.082	0.600	0.940	0.1446
VR	Training	0.828	0.828	0.506	0.298	82.754	0.086	0.700	0.887	
	Testing	0.936	0.999	0.453	0.299	93.606	0.066	0.740	0.940	0.1369
CB	Training	0.828	0.828	0.506	0.298	82.754	0.087	0.700	0.887	
	Testing	0.937	0.999	0.453	0.299	93.606	0.067	0.740	0.940	0.1369
KNN	Training	0.713	0.713	0.653	0.485	71.396	0.118	0.407	0.700	
	Testing	0.669	0.796	1.031	0.621	67.288	0.175	0.420	0.700	0.1913
GB	Training	0.734	0.734	0.628	0.400	73.420	0.113	0.553	0.813	
	Testing	0.677	0.807	1.018	0.538	68.109	0.172	0.620	0.820	0.1897
RF	Training	0.753	0.753	0.606	0.399	75.371	0.107	0.567	0.833	
	Testing	0.702	0.832	0.978	0.591	70.517	0.163	0.560	0.720	0.1852
ANN	Training	0.710	0.710	0.656	0.402	71.225	0.119	0.633	0.833	
	Testing	0.623	0.256	1.100	0.548	63.802	0.192	0.640	0.800	0.1997
RR	Training	0.569	0.569	0.800	0.480	57.054	0.158	0.533	0.780	
	Testing	0.634	0.726	1.083	0.574	63.992	0.188	0.580	0.800	0.1976
LR1	Training	0.407	0.407	0.938	0.596	40.701	0.207	0.347	0.707	
	Testing	0.318	0.968	1.479	0.860	31.863	0.318	0.300	0.740	0.2628

### Performance comparison using visualization

#### Regression error characteristic curve

**Figure 19:**
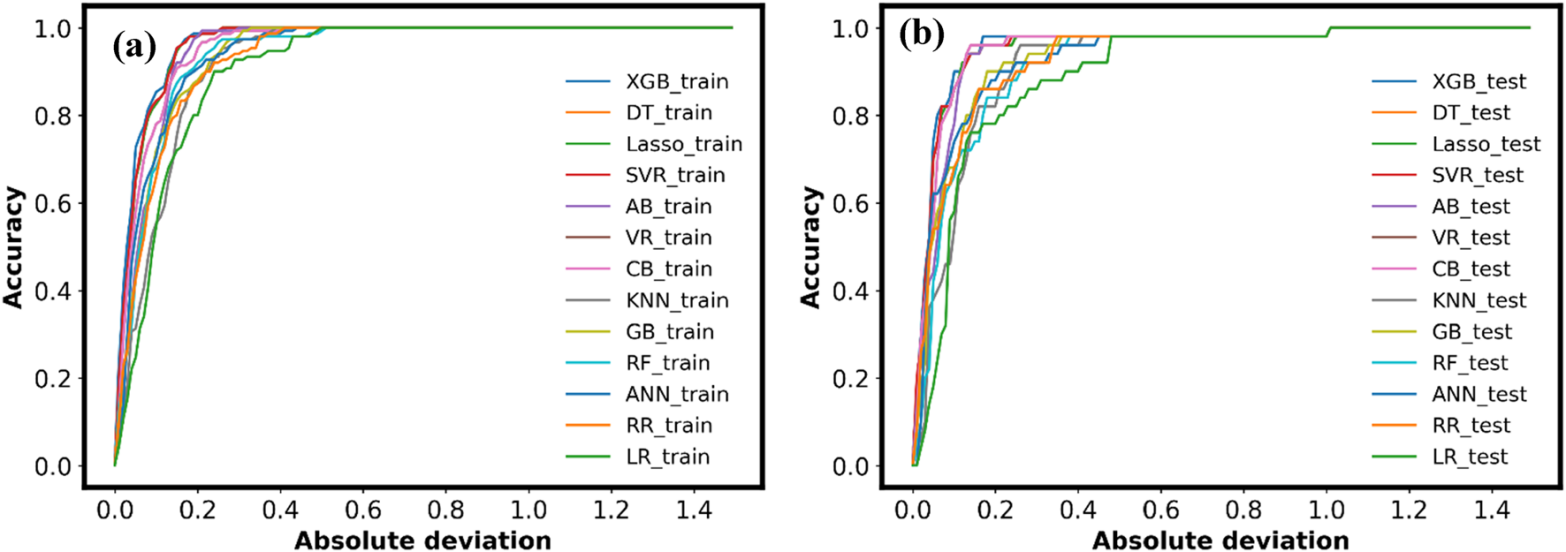
Regression error characteristic curve for employed models. (**a**) training phase (**b**) testing phase.

In classification tasks, the receiver operating characteristic (ROC) curve is widely used as a useful tool for comparing and displaying classification results. Regression error characteristic (REC) curves are developed in the domain of regression for similar purpose as ROC^102–105^. The proportion of correctly predicted occurrences within a certain tolerance interval (y-axis) are shown versus the absolute deviation tolerance (x-axis) in REC curves. This curve successfully shows the cumulative distribution function of prediction errors either represented in absolute deviation or squared residual terms. Area under curve (AUC) may be used to calculate the area over the REC Curve (AOC), which is a skewed estimate of anticipated errors using the formula AOC = 1-AUC. An ideal regression model would have a curve that is parallel to the y-axis and an AOC that is as small as possible. The error is displayed using absolute deviation as shown in Fig. 19(a) and (b) for the training and testing datasets, respectively. AOC values for several machine learning models are shown in Table 6. The significantly smaller AOC values here represent better model performance. The XGB model performs exceptionally well during training (AOC = 0.038) and testing (AOC = 0.048).

**Table 6:** Results of REC curve.

Phase\Model	XGB	DT	LR2	SVR	AB	VR	CB	KNN	GB	RF	ANN	RR	LR1
Training	0.038	0.041	0.041	0.041	0.054	0.049	0.049	0.077	0.063	0.063	0.061	0.069	0.089
Testing	0.048	0.051	0.051	0.051	0.059	0.053	0.053	0.087	0.072	0.085	0.073	0.075	0.107

#### Taylor Diagram

**Figure 20:**
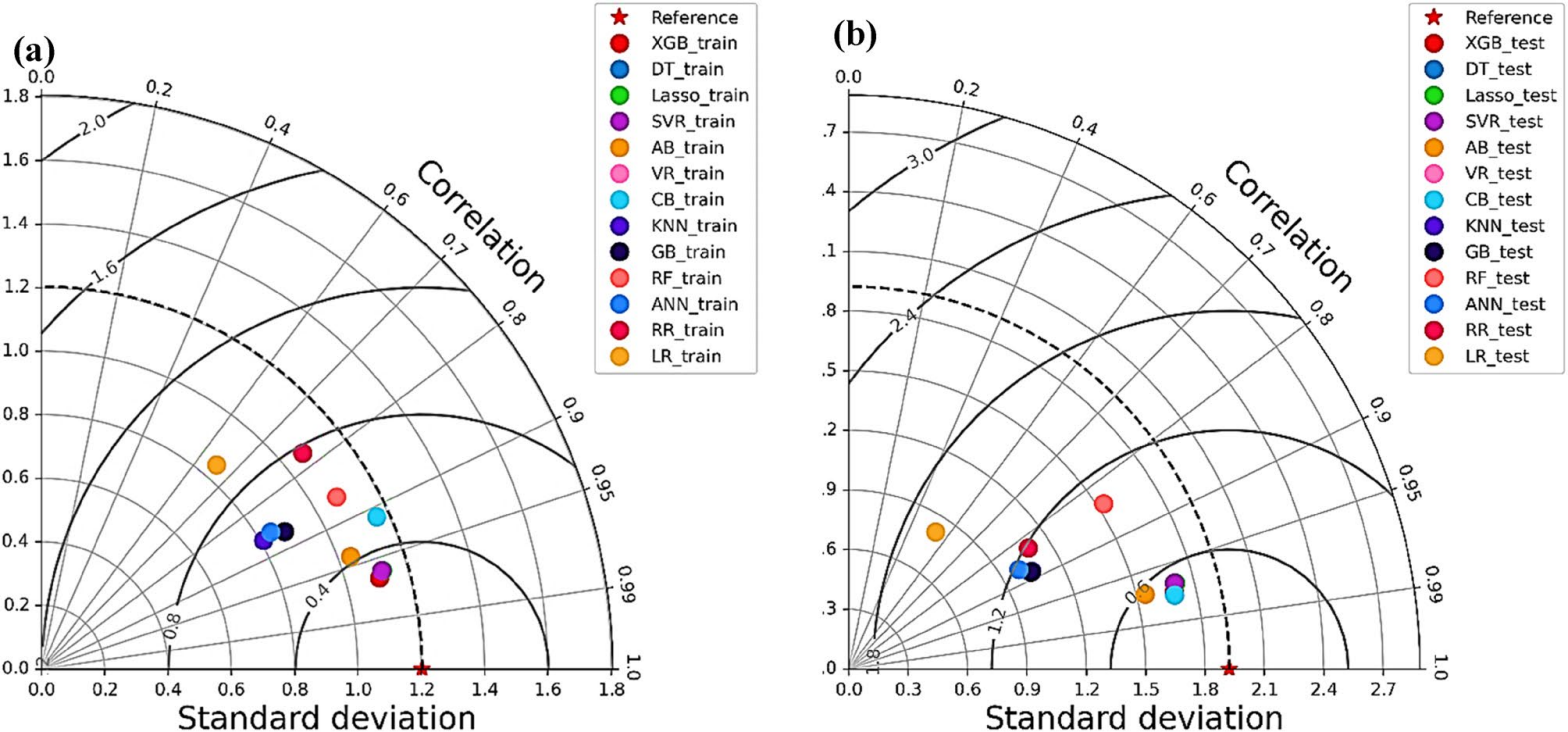
Taylor diagram (**a**) Training data (**b**) Test data.

Taylor diagram was originally developed by Karl E. Taylor for a comparative analysis of the performance metrics of the models^106^. This diagram incorporates three primary metrics SD, R, and RMSE. R and SD are used to measure how well the models' predictions align with experimental data, whereas RMSE quantifies the discrepancies between predicted and actual values. An optimal model is indicated by R values close to 1 and an SD that mirrors the experimental data's SD. The Taylor diagram serves as an effective tool for assessing model accuracy by illustrating the model that best aligns with the actual data. By displaying multiple models or datasets on the same graph, it facilitates a direct comparison, allowing for an evaluation of their correlation, variance, and RMSE in relation to the reference data (actual dataset)^106,107^. In Fig. 20**,** the proximity of XGB, decision tree and lasso regression model to the reference point on this diagram underscores its remarkable accuracy in prediction. On the other hand, linear regression and ridge regression are further away from reference showing their lower performance.

#### Score analysis

**Figure 21:**
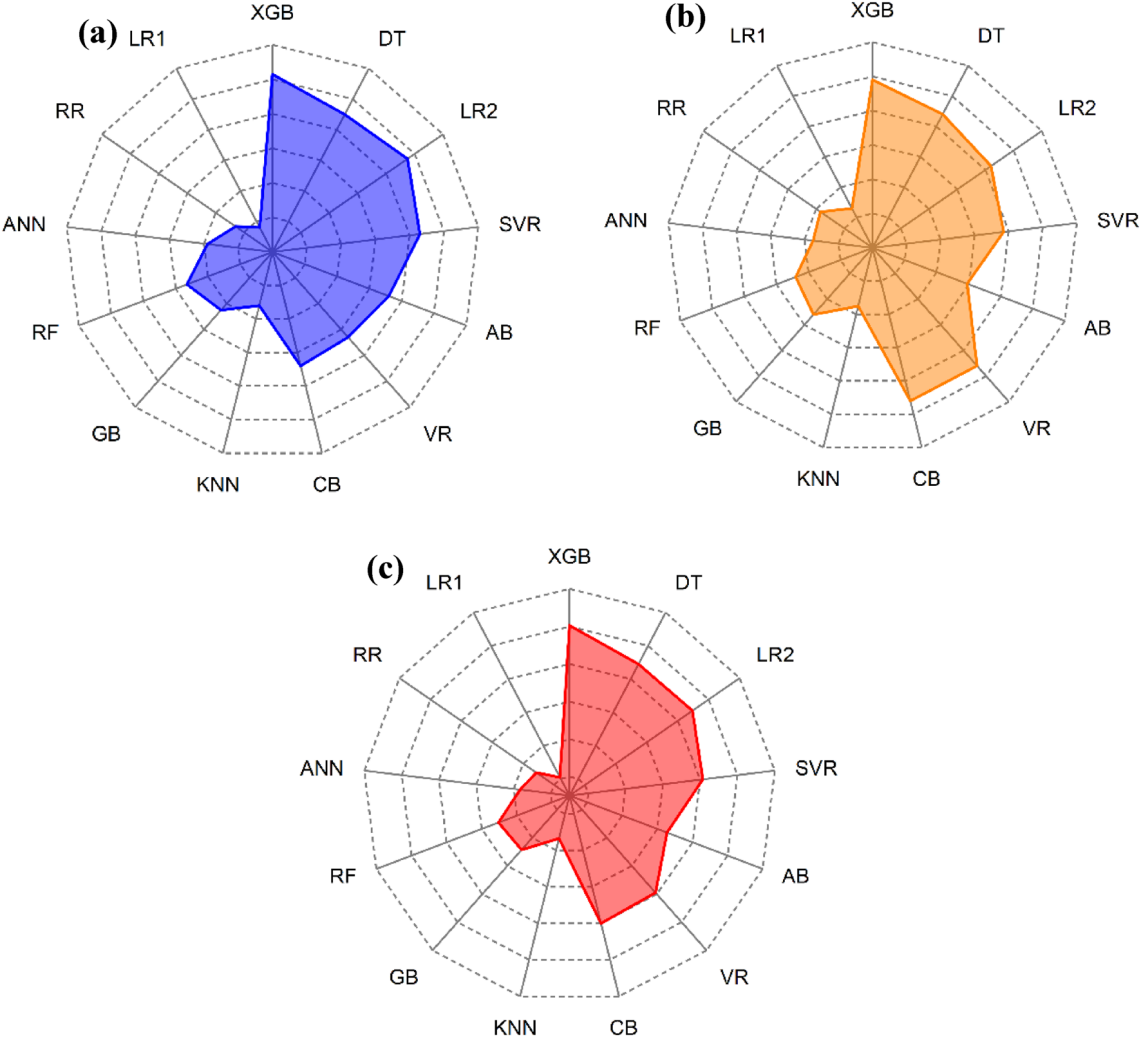
Score analysis results (**a**) training phase (**b**) test phase (**c**) Total.

The Score Analysis method makes it easy to evaluate and compare performance of employed models and visualize the top performing models. The models in this analysis are sorted according to performance metrics, with the best-performing model receiving the highest rank and the worst-performing model receiving the lowest. The total number of models (k) in this study is 13. The training and test parts of this ranking procedure are carried out independently. The total of the test and training phase scores determines the final score. Table 7 presents a comprehensive overview of the score analysis, while Fig. 21 presents a radar map that illustrates the outcomes. Upon analyzing the table and figure, XGB and DT were the two best models throughout training, with scores of 103 and 90, respectively. Their performance decreased during the test phase with XGB scoring 98 and DT scoring 88.

**Table 7:** Score analysis of model performances.

Model	Phase	R^2^	NSE	RMSE	MAE	VAF%	PI	a10	a20	Phase total	Total
XGB	Train	13	13	13	13	13	13	13	12	103	201
	Test	13	8	13	13	13	13	13	12	98	
DT	Train	11	11	11	11	11	11	11	13	90	178
	Test	10	10	10	12	10	10	13	13	88	
LR2	Train	12	12	12	11	12	12	11	13	95	179
	Test	9	11	9	11	9	9	13	13	84	
SVR	Train	10	10	10	12	10	10	12	12	86	163
	Test	8	9	8	10	8	8	13	13	77	
AB	Train	9	9	9	8	9	9	8	11	72	131
	Test	7	2	7	7	7	7	9	13	59	
VR	Train	7	7	7	10	7	8	10	10	66	158
	Test	11	12	12	9	11	12	12	13	92	
CB	Train	8	8	8	9	8	7	10	10	68	160
	Test	12	13	11	8	12	11	12	13	92	
KNN	Train	4	4	4	3	4	4	4	5	32	67
	Test	4	4	4	2	4	4	6	7	35	
GB	Train	5	5	5	6	5	5	6	8	45	97
	Test	5	5	5	6	5	5	10	11	52	
RF	Train	6	6	6	7	6	6	7	9	53	101
	Test	6	6	6	3	6	6	7	8	48	
ANN	Train	3	3	3	5	3	3	9	9	38	73
	Test	2	1	2	5	2	2	11	10	35	
RR	Train	2	2	2	4	2	2	5	7	26	63
	Test	3	3	3	4	3	3	8	10	37	
LR1	Train	1	1	1	2	1	1	3	6	16	42
	Test	1	7	1	1	1	1	5	9	26

## Feature importance

**Figure 22:**
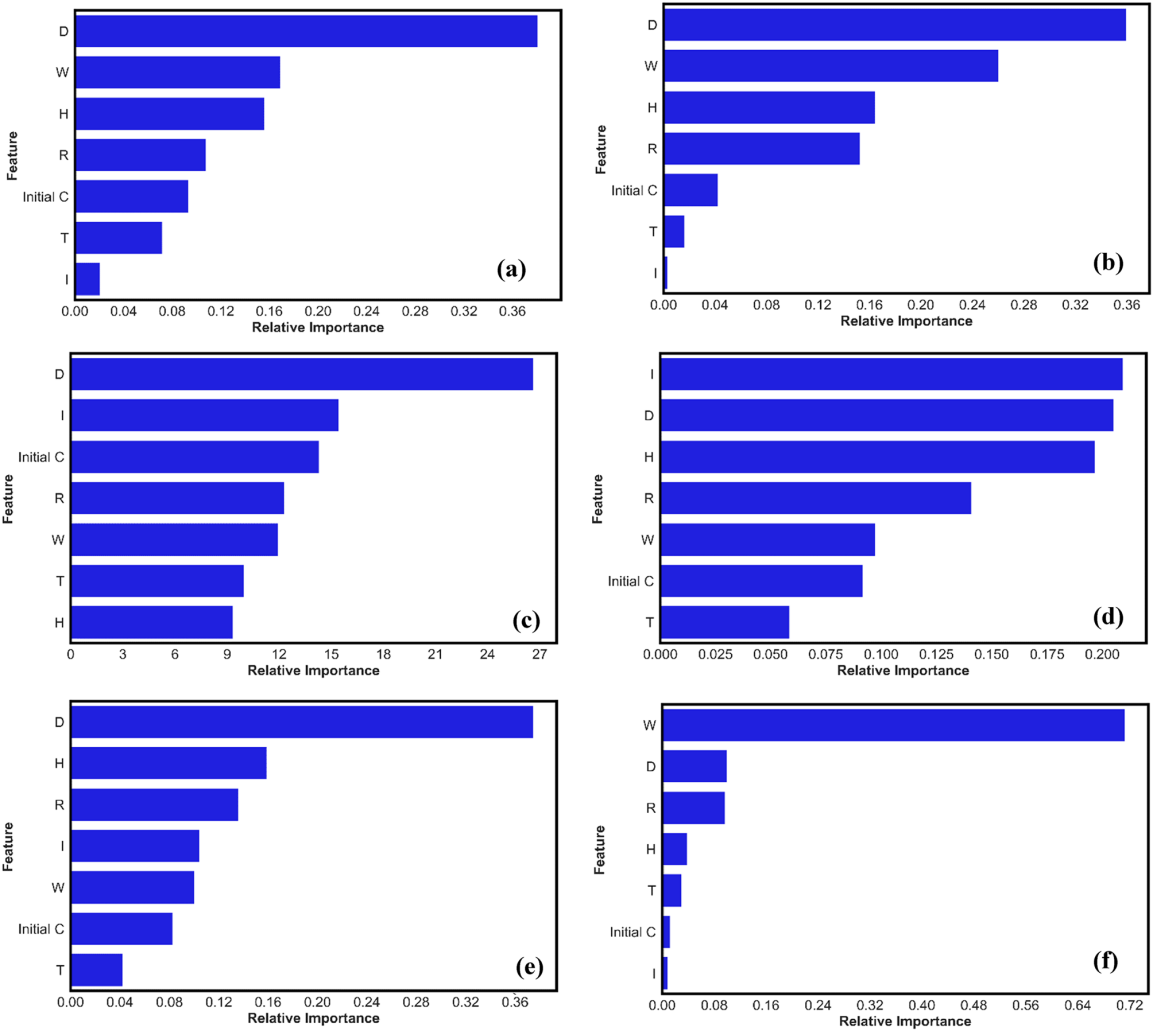
Feature importance of employed models (**a**) GB (**b**) Decision tree (**c**) CatBoost (**d**) AdaBoost (**e**) RF (**f**) XGB.

The emerging field of explainable AI (XAI) brings transparency and help users understand decision making process of ML model. The effort addresses the issue of "AI black boxes," or systems where core operations are opaque, keeping engineers and practitioners in the dark about results. Researchers and engineers push for openness in ML system operation, opposing such opacity. XAI aims to reveal the reasoning behind an ML's decisions by removing layers of complexity. Employing feature importances in this study will reveal role of input parameters in final predictions. The absolute significance of each input parameter in the suggested models of GB, Decision tree, CatBoost, AdaBoost, RF, XGB is depicted in Fig. 22(a)–(f). The most important feature for most models for estimating the photocatalytic degradation rate is D for GB, DT, CB, and RF while for AB is I and XGB is W. The significant influence of D, W and I on the photocatalytic degradation rate is seen in all the top-performing models. Notably, the XGB model (Fig. 22(f) emphasizes the importance of W, D and R as a critical component of photocatalytic degradation rate. In RF, GB, AB, DT and CB, Dosage remains the most important feature with 35%, 38.5%, 29.6%, 35.9 and 26.6% percentage weightage While in XGB model W remain most important with 71% weightage. This will also have a physical importance as interpretable machine learning models provide light on the underlying mechanisms and demonstrate their potential as useful instruments for forecasting and identifying critical variables influencing the TiO_2_ photocatalytic degradation rate. Pervious researchers have performed experimental techniques to assess the role of input perimeters and experimental settings on final output. Haghighatmamaghani et al.^108^ researched the operating parameters of concentration, relative humidity level, and residence time on removal efficiency and maintained that humidity conditions play an important role. Zhang et al.^109^ researched the humidity influence on photocatalytic degradation and tried to develop a relationship. Li et al.^32^ used TiO_2_ photocatalyst and concluded that the degradation of organic pollutants was influenced by several factors, including the dosage of TiO_2_, initial solution pH, duration of UV light irradiation, and solution temperature. Similarly, Schossler et al.^110^ used SHapley Additive exPlanations (SHAP) technique to interpret model predictions and reveal role of input parameters.

## Conclusions

In this study, thirteen common ML models are utilized to examine an extensive dataset and predict Photocatalytic degradation rate of TiO_2_. A variety of ML techniques are used in this including linear approaches (such as linear, ridge, lasso, and support vector regression), decision trees, random forests, K-nearest neighbors, and boosting-based methods. All models are trained, tested using the given input parameters and rigorously evaluated. Feature importances are utilized to reveal underlying physical phenomena and decision-making process. The results obtained are the following.

- XGB model has the highest R^2^ value of 0.932 and 0.937 in training and test phase. The DT, LR2 and SVR model showed good performance with R^2^ value of 0.927 in training phase and 0.924, 0.924 and 0.923 in test phase respectively. While ANN, RR and LR1 showed lowest R^2^ value of 0.62, 0.63 and 0.31 in training phase and 0.70, 0.56 and 0.40 in test phase respectively. AB, VR and CB models gave average performance with R^2^ values of 0.86, 0.86 and 0.82 in training and 0.90,0.93 and 0.93 in test phase. These models’ performance significantly improved in the test phase.
- After regression and statistical analysis, Regression error characteristics were used to assess and visualize model performances. XGB, DT model has the least AOC values of 0.037 and 0.041 in training and 0.04 and 0.051 in test phase.
- XGB, DT, LR2 achieved the highest score in training 103, 90,95 respectively and 98, 88 and 84 respectively in test phase. ANN, RF and LR1 showed lowest scores of 38, 26 and 16 in training and 35, 57 and 26 in testing phase.
- The influence of D, W, and I on photocatalytic degradation rate is consistent across all top-performing models. The most crucial feature for estimating photocatalytic degradation rate varies among different models, with D being significant for GB, DT, CB, and RF, and I for AB, while W is important for XGB.
- XGB, DT, LR2 are the most robust ML models and are prediction problem of TiO_2_ photocatalytic degradation of air contaminants.

In summary, this research study extensively evaluated thirteen ML techniques for estimation of TiO_2_ photocatalytic degradation of air contaminants. This study successfully proposed the most robust XGB, DT and LR2 models for prediction. Moreover, it is essential to recognize the limitations of the study and recommendations for future work. The developed ML models are trained for database input parameters, necessitating additional training, testing, and hyperparameter tuning for new inputs. More experimental factors should be studied to enhance the diversity and applicability of the models and study their role in TiO_2_ photocatalytic activity. New machine learning techniques should also be used for the analysis of the present database to evaluate the impact of input parameters and model’s performance. The ML models that have been constructed can also be used for prediction in various engineering problems.
