# Publisher Correction: Evaluation of machine learning models for predicting TiO_2_ photocatalytic degradation of air contaminants

**DOI:** 10.1038/s41598-024-66716-4

**Published:** 2024-07-11

**Authors:** Muhammad Faisal Javed, Muhammad Zubair Shahab, Usama Asif, Taoufik Najeh, Fahid Aslam, Mujahid Ali, Inamullah Khan

**Affiliations:** 1 Department of Civil Engineering, Ghulam Ishaq Khan Institute of Engineering Sciences and Technology, Topi, Pakistan; 2 Western Caspian University, Baku, Azerbaijan; 3 COMSATS University Islamabad, Abbottabad Campus, Abbottabad, Pakistan; 4 Department of Civil Engineering, Nazarbayev University, Astana, Kazakhstan; 5 Operation and Maintenance, Operation, Maintenance and Acoustics, Department of Civil, Environmental and Natural Resources Engineering, Lulea University of Technology, Luleå, Sweden; 6 Department of Civil Engineering, College of Engineering in Al-Kharj, Prince Sattam Bin Abdulaziz University, 11942 Al-Kharj, Saudi Arabia; 7 Department of Transport Systems, Traffic Engineering and Logistics, Faculty of Transport and Aviation Engineering, Silesian University of Technology, Krasińskiego 8 Street, 40-019 Katowice, Poland; 8 National Institute of Transportation, National University of Sciences and Technology (NUST), Islamabad, Pakistan

Correction to: *Scientific Reports* 10.1038/s41598-024-64486-7, published online 13 June 2024

The original version of this Article contained errors in the Affiliations.

Muhammad Faisal Javed was incorrectly affiliated with ‘National Institute of Transportation, National University of Sciences and Technology (NUST), Islamabad, Pakistan’.

As a result, the affiliation tagging in the author list was incorrect. The correct affiliations for all authors are listed below.

**Muhammad Faisal Javed**

Department of Civil Engineering, Ghulam Ishaq Khan Institute of Engineering Sciences and Technology, Topi, Pakistan

Western Caspian University, Baku, Azerbaijan

**Muhammad Zubair Shahab**

COMSATS University Islamabad, Abbottabad Campus, Abbottabad, Pakistan

**Usama Asif**

Department of Civil Engineering, Nazarbayev University, Astana, Kazakhstan

**Taoufik Najeh**

Operation and Maintenance, Operation, Maintenance and Acoustics, Department of Civil, Environmental and Natural Resources Engineering, Lulea University of Technology, Luleå, Sweden

**Fahid Aslam**

Department of Civil Engineering, College of Engineering in Al-Kharj, Prince Sattam Bin Abdulaziz University, 11942 Al-Kharj, Saudi Arabia

**Mujahid Ali**

Department of Transport Systems, Traffic Engineering and Logistics, Faculty of Transport and Aviation Engineering, Silesian University of Technology, Krasińskiego 8 Street, 40-019 Katowice, Poland

**Inamullah Khan**

National Institute of Transportation, National University of Sciences and Technology (NUST), Islamabad, Pakistan

The original Article has been corrected.

